# Factors Determining Quality of Drug Information by Hospital Pharmacies—Results from Five-Year Annual Quality Assessment

**DOI:** 10.3390/pharmacy12040109

**Published:** 2024-07-13

**Authors:** Dorothea Strobach, Ute Chiriac, Sigrun Klausner, Sabine Krebs, Claudia Langebrake, Christiane Querbach, Carolin Schuhmacher, Rickmer Schulte, Simon Wiegrebe, Ute Amann

**Affiliations:** 1 Hospital Pharmacy, University Hospital, LMU Munich, Marchioninistr. 15, 81377 Munich, Germany; 2 Doctoral Program Clinical Pharmacy, University Hospital, LMU Munich, Marchioninistr. 15, 81377 Munich, Germany; 3 Department of Pharmacy, University Hospital of Heidelberg, Im Neuenheimer Feld 670, 69120 Heidelberg, Germany; ute.chiriac@med.uni-heidelberg.de; 4 Landesapotheke Salzburg, Muellner Hauptstr. 50, 5020 Salzburg, Austria; si.klausner@salk.at; 5 Pharmacy Department, Erlangen University Hospital, Palmsanlage 3, 91054 Erlangen, Germany; sabine.krebs@uk-erlangen.de; 6 Hospital Pharmacy, University Medical Center Hamburg-Eppendorf, Martinistraße 52, 20246 Hamburg, Germany; c.langebrake@uke.de; 7 Department of Stem Cell Transplantation, University Medical Center Hamburg-Eppendorf, Martinistraße 52, 20246 Hamburg, Germany; 8 Hospital Pharmacy Department, Klinikum Rechts der Isar, Technische Universität Munich, Ismaninger Straße 22, 81675 Munich, Germany; christiane.querbach@mri.tum.de; 9 Hospital Pharmacy, Schwarzwald-Baar Hospital Villingen-Schwenningen, Klinikstr. 11, 78050 Villingen-Schwenningen, Germany; carolin.schuhmacher@sbk-vs.de; 10 Statistical Consulting Unit StaBLab, Department of Statistics, LMU Munich, Ludwigsstraße 33, 80539 Munich, Germany; rickmer.schulte@stat.uni-muenchen.de (R.S.); simon.wiegrebe@stat.uni-muenchen.de (S.W.); 11 Faculty of Medicine at LMU Munich, 80336 Munich, Germany; ute.amann@lmu.de

**Keywords:** drug information, hospital pharmacy, quality assessment

## Abstract

Drug information (DI) provided by hospital pharmacies aims to promote rational and safe drug therapy. While quality assessment for this task is recommended, more knowledge on the factors determining the quality is needed. We aimed to evaluate the impacts of different factors on the quality of DI provided by hospital pharmacies to healthcare professionals. Retrospectively, answers on fictitious enquiries about annual DI tests for German hospital pharmacies over five years were evaluated for content-related and structural requirements. Multivariate analysis was performed for the impact of the enquiry complexity, DI organization (specialized DI center; pharmacist responsible per day; DI on top of other routine tasks), and quality measures (second look; experience of answering pharmacist in DI/on ward; use of documentation database). In 2017–2021, 45, 71, 79, 118, and 122 hospital pharmacies participated. The enquiry complexity had a statistically significant impact on the content-related quality, with poor results for a higher complexity (years 2018/2021, OR 0.25/0.04, *p* < 0.01). The DI centers achieved better results regarding content-related quality than for a pharmacist responsible per day (OR 0.76/*p* = 0.65) or DI on top of routine tasks (OR 0.35/*p* = 0.02). The DI centers scored better in structural quality. The second look showed an overall trend of a better content-related and structural quality. In conclusion, specialized DI centers and second looks are recommended as quality-improving measures. Training for answering complex enquiries should be intensified.

## 1. Introduction

The World Health Organization recognizes independent drug information (DI) as a core component for the rational and effective use of drugs [1]. The information must be available to health practitioners in a suitable form and be of relevance to the current clinical practice [2]. DI, as a professional task provided to healthcare practitioners, should be unbiased, evidence-based, usable, and timely to improve medication safety [2,3,4]. This requires professional skills in searching and abstracting the literature, the interpretation of data for specific clinical situations, and the discussion of possible solutions with the enquirer [2,5]. Pharmacists play a major role in this task. In some countries, like the United Kingdom (UK) and Norway, DI services working nationwide or for defined areas are implemented [6,7]. In Germany, regulatory requirements demand that every hospital pharmacy has to provide DI to the medical staff of their hospital [8]. Compared to other European countries, the number of hospital pharmacists in Germany is low, with 0.4 per 100 hospital beds [9]. Thus, depending on the hospital size and staffing of the pharmacy, the organization of DI as a professional task differs substantially. While some hospital pharmacies have a specialized DI center, others have to manage DI enquiries on top of their daily tasks in dispensing or compounding. However, similar situations can be found in other countries.

International practice guidelines on the provision of DI demand regular quality assurance procedures [2,3,4,10,11]. In 2017, the DI working group of the German association of hospital pharmacists (ADKA e.V.) started an annual quality assessment for DI provided by hospital pharmacies using a fictitious enquiry and simulated real-life conditions. Answers are evaluated for content-related and structural (formatting and presentation of information) requirements. The approach has been validated to reliably reflect DI quality in comparison to real enquiries answered by participating hospital pharmacies [12].

While a variety of studies have evaluated the satisfaction of enquirers and the clinical or economic impact of DI provided by pharmacists [13,14,15], only a few studies have assessed factors possibly impacting the quality of DI. The aspects of quality cover the content and structure of an answer. Content-related aspects refer to the correct answering of the enquiry, including all relevant information to handle the clinical problem and the absence of wrong and misleading information [2,3,4,10,16]. Requirements concerning the structure of an answer refer to the format, how the information is presented, and will have a great impact on the readability and understanding of the answer [2,3,4,10,17,18]. As an example, a study evaluating written answers on DI enquiries identified specific advice and giving a conclusion as positive factors, while unexplained abbreviations and study findings had a negative impact [18].

Factors with an impact on the quality of DI can be related to the process of answering DI enquiries as defined by guidelines. The important steps of this process are the correct assessment of the enquiry, including all relevant background information and assignment to a DI topic, an appropriate search strategy, the formulation of an answer adjusted to the enquirers’ needs, documentation, and follow-up [2,3,4,10]. For example, insufficient assessments of background information led to incorrect responses and inappropriate recommendations [19]. Additionally, the topic and complexity of enquiries could be of concern. While several studies have described how the enquiry complexity will influence the time required to answer it, its impact on the quality of answers has not been addressed so far [20,21,22,23]. Moreover, the organizational aspects of DI as a professional task in hospital pharmacies might have an impact, e.g., the presence of a special DI center or the professional experience of the answering pharmacist. In the first quality assessment for German hospital pharmacies, participants with a DI center achieved better results than participants where pharmacists had to answer the enquiry in addition to other daily tasks [12]. Studies that focused on the time necessary to answer DI enquiries found that staff members with less than one year of experience needed significantly more time [24]. However, there was only a weak association between time consumption and the quality of responses in a further study [22]. The experience of the answering pharmacist in DI, as well as background knowledge as a ward pharmacist, might have a pronounced impact on the quality of an answer. A second look procedure, a second pharmacist reviewing the prepared answer, and the use of a documentation system for enquiries and answers are recommended by guidelines on the provision of DI as quality assurance instruments [2,3,4]; however, their impact on the quality of DI has not been thoroughly studied. The aim of our study was to broaden the evidence on factors possibly influencing the quality of DI provided by hospital pharmacies to healthcare professionals. Therefore, data of the German annual DI quality assessment of five years were analyzed regarding the impacts of different factors on the quality of DI responses.

## 2. Materials and Methods

### 2.1. Annual DI Quality Assessment

The DI working group of ADKA have organized a voluntary annual quality assessment of DI since 2017 [12]. Hospital pharmacies are invited to participate via the mailing list of ADKA. The setting is non-blinded, as blinding to the test enquiry is not possible, since normally, only enquiries from their own hospital will be answered by hospital pharmacies. Instead, for simulated real-life conditions, participants were only given the test week, but not the exact day, and a time frame for answering adjusted to the complexity of the enquiry was set. Upon registration, the hospital pharmacies were given a consecutively created number of participation. On the test day, a fictitious enquiry was sent via e-mail. Answers had to be sent anonymously, but displaying the number of participation.

In advance, a fictitious enquiry with yearly changing topics and content-related requirements was defined by an expert panel of seven DI pharmacists. Content-related requirements were divided into essential (crucial for answering the enquiry) and optional (additional beneficial information for the enquirer). Structural requirements were defined based on the literature (Table 1) [17,18]. One expert served as an unblinded communicating pharmacist. Three blinded experts (allocated via block randomization, where the same experts did not judge all the same answers) rated all answers separately for all predefined content-related and structural requirements as fulfilled (1) or not (0). The communicating pharmacist summarized all expert ratings.

The participating hospital pharmacies received feedback on their fulfilment of the requirements. In addition, a questionnaire was sent to all participants on the characteristics of the hospital pharmacy, asking for the number of pharmacists, organization of the DI service (DI center; pharmacist responsible per day; DI in addition on routine tasks), implemented DI quality measures (second look; answering pharmacist has experience on ward; use of a DI documentation system; additional measures), and clinical experience of the pharmacist who actually answered the test enquiry (level 1: <1 year; level 2: 1–3 years; level 3: 3–5 years; level 4: >5 years).

### 2.2. Data Collection

The characteristics of the fictitious enquiries in 2017–2021 were assessed for topic and the number of essential and optional content-related requirements. The test enquiries were categorized by the expert panel according to the UK Medicines Information (UKMI) Enquiry Level [25]: Level 1 (simple enquiries; answered from one or two standard sources), Level 2 (complex enquiries; use of multiple/more specialist sources; available evidence provides a reasonably clear answer/course of action), and Level 3 (complex enquiries; absence of a clear answer/course of action, professional judgement needed; multiple sources/evaluation of primary literature.)

Data on the results of the experts’ ratings concerning content-related requirements and the presence of irrelevant information in the answers were assessed. For structural requirements, the number achieved out of ten predefined requirements was determined. For detailed analysis, ratings concerning the presentation of references, presentation of references in a way to be tracked or checked, and presentation of a conclusion were documented.

### 2.3. Statistical Analysis

Data documentation was conducted with Microsoft Excel^®^ 2016 (Seattle, WA, USA). Continuous variables are presented as median and range (min/max), and categorical variables as frequency distribution. Statistical analyses were performed using IBM SPSS Statistics^®^ version 25.0 (Armonk, NY, USA) and, for multivariate logistic regression, with R (version 4.2.2, R Foundation for Statistical Computing, Vienna, Austria).

Multivariate logistic regression models were performed for the influence of organizational and structural parameters on the quality of the DI responses, and Odds Ratios (ORs) and 95% confidence intervals (CI) were calculated.

Quality regarding the content was tested in two ways: as the presence of all essential information in an answer and, in addition, as the percentage of essential information fulfilled. For the percentage of essential information fulfilled, depending on the number of essential information per year (2, 3, or 5), the possible values could be 0.33, 0.5, 0.66, or 1.

Parameters tested for their impact on the content-related quality of the DI answers were UKMI enquiry level (as the test year), DI organization, and the number of pharmacists of the hospital pharmacy. The experience of the answering pharmacist in DI, their experience on ward, second look, and the use of a documentation system were tested as quality measurements. The intercept was the estimate for year 2017, with organization as a DI center and zero for number of pharmacists, experience in DI, second look, experience on ward, and documentation system.

For quality regarding structural requirements, the same characteristics were tested separately for “references given”, “references given in a trackable way”, and “conclusion presented”.

The outcome variables exhibited only weak pairwise correlations, hence, all were analyzed separately. A statistical significance level of α = 0.05 was used.

### 2.4. Ethics Approval

Ethics approval was not obtained and is not necessary according to the general information provided by the ethics committee of the medical faculty of the LMU Munich, since no patient-specific data, only fictitious cases, and no personal data on the responding pharmacists were documented or analyzed. Only retrospective irreversible anonymized data were used in the secondary analysis

## 3. Results

### 3.1. Characteristics of Annual Assessment

Table 2 shows the topic of the fictious enquiry, UKMI enquiry level, number of predefined essential and additional useful information per year. The test year is closely related to the enquiry complexity (UKMI enquiry level). The full test enquiries and expected predefined essential information are presented in Supplementary Table S1 [12,26,27,28,29].

Table 3 shows the number and characteristics of the participating hospital pharmacies and results on content-related and structural requirements. Regarding the organization of DI, in most hospital pharmacies, DI was performed on top of routine tasks (70% over all five years). Regarding content-related results, the percentage of participants with all essential information varied widely depending on the test year from 7% to 94%. We also tested for irrelevant information in the answers, which was present in about a quarter of the answers, and in the year 2020, even in 45%. Additionally, the presence of misleading or wrong information was analyzed. This was the case in 12–18% of the answers, and in the year 2019, even in 44%. For structural requirements, the median number achieved increased over the years. Further analysis of selected structural requirements showed an increase in the correct presentation of references and a conclusion/recommendation over the years.

### 3.2. Multivariate Logistic Regression on the Influence of Parameters on Content-Related Quality of Answers

Table 4, part A presents results for quality defined as the presentation of all essential information in the answer. A strong effect on this outcome variable was found for the variable year. In 2018 and 2021, the ORs of presenting all essential information were 0.25 and 0.04, respectively. In both years, the enquiry level was 3 and the number of essential information was five. In contrast, in 2017, 2019, and 2020, the odds of presenting all essential information were high (4.28, 8.19, and 1.99, respectively). In these years, the enquiry level was 2 and the number of essential information was two or three. These results point to an impact of topic and enquiry complexity on the quality of the answers. Furthermore, we observed an impact of DI organization. In comparison to the presence of a DI center in the hospital pharmacy, organization as a pharmacist responsible per day (OR 0.74; *p* = 0.65) or DI in addition to routine tasks (OR 0.35; *p* = 0.02) had a negative impact.

In part B of Table 4, the quality of DI answers was considered as the percentage of essential information fulfilled. Regarding the impact of the test year and DI organization, the results were comparable to part A of the table.

The number of pharmacists in the hospital pharmacy and all quality measures seemed to have no impact on the two outcome variables considered above.

In addition, we tested the influence of the described parameters on the presence of irrelevant and misleading or wrong information as being of negative quality. Concerning irrelevant information, no parameter had a statistically significant impact, with the exception of the year 2020. Regarding misleading or wrong information, the only statistically significant impact was found for the year 2019, where the odds were high.

### 3.3. Multivariate Logistic Regression on the Influence of Parameters on Structural Quality of Answers

We tested the same parameters as for content-related quality on their impact on the structural quality of answers. Tests were performed for three selected structural requirements (Table 5). For all three, an increase in the odds of fulfillment was found over the years. Regarding DI organization, a trend to a negative impact on all three structural requirements could be seen for organization as a pharmacist responsible per day or DI in addition to routine tasks compared to a DI center. Second look had a positive impact on the odds of presenting references in a trackable way (OR 1.92; *p* = 0.01). A positive trend for second look can also be seen in the presentation of references and a conclusion, although this was not statistically significant.

## 4. Discussion

In this retrospective analysis, we evaluated the impact of process-related and structural factors on the quality of DI provided by hospital pharmacies. In a unique setting, we were able to analyze the answers to DI test enquiries from repeated annual tests over five years with predefined content-related and structural requirements, taking into account the enquiry level, organization of the task of DI in the hospital pharmacy, and implemented quality measures. Our evaluations showed that the enquiry complexity, resembled by the variable year, had a significant impact on the content-related quality of the DI answer, with poorer results for more complex enquiries. Moreover, the organization of DI had a clear impact on the content-related quality. Compared to hospital pharmacies with a DI center, poorer results were achieved if a pharmacist was responsible per day or DI had to be performed on top of other routine tasks. This was independent of the overall number of pharmacists working in the hospital pharmacy. The analysis of the quality of the DI answer regarding structural requirements revealed a general positive trend over the years, which possibly was a learning effect from the repeated annual tests. Again, the presence of a DI center achieved better results than other organizational forms. Surprisingly, there was hardly any impact of the evaluated quality measures on content-related and structural quality, with the exception of a second look procedure.

Drug information enquiries differ substantially regarding their complexity, the effort needed in the literature search, and the interpretation of data, as reflected by the UKMI enquiry level. The test enquiries were categorized as level 2 or 3, with higher numbers of predefined essential information for level 3 enquiries. The proportion of hospital pharmacies presenting all expected essential information was clearly lower for these years. The effect of the test year, related to the enquiry complexity and topic, proved to be a factor impacting the quality of DI in multivariate analysis. Especially in 2021, the results were poor. We think that, in addition to the enquiry level, the particular topic was responsible. Enquiries on adverse drug reactions (ADRs), the general topic of 2021, are one of the major topics in DI and answering them should be routine [20,30,31,32,33,34]. However, the test enquiry in 2021 specifically asked for ADRs on male fertility, a topic which might be unfamiliar to participants with a poor background knowledge. In particular, pharmacists might have poor knowledge on the complex hormonal regulation of spermatogenesis and male sexual function. Indeed, the impact of drugs on male fertility is rather neglected. For instance, in a retrospective study, 47% of men with an unfulfilled wish to father a child took drugs, with 16% of those with a known fertility impairment and 51% with an impairment of male sexual function [35].

The quality of DI is also determined by the addition of further helpful information, which is not asked for specifically, and the absence of wrong, misleading or irrelevant information. For the annual test enquiries, the number of predefined optional contents as additional helpful information differed depending on the specific topic. Most participants included the optional content in their answer. Unfortunately, irrelevant information concerned a quarter of all answers. This was independent of the test year, with the exception of 2020, where the odds more than doubled. The topic in this year was overdose in a geriatric patient. Despite a specific enquiry was asked by a geriatrician, who expected to critically evaluate drug therapy of elderly people, many participants performed a detailed medication analysis for a geriatric patient. Moreover, misleading or wrong information was part of 12–18% of the answers, and in 2019, even in 44%. These are disappointing results, and while irrelevant information is simply annoying, considering time as valuable resource for health practitioners, wrong or misleading information could be a hazard for drug safety. In this study, we did not further evaluate if patient harm would have been likely based on the wrong or misleading information. One would expect that quality measures like second look would decrease the probability of including irrelevant, wrong, and misleading information. Unfortunately, in the multivariate analysis, no effect was seen. The organization of DI also had no impact in this regard. Incorrect answers on DI enquiries have also been found in previous studies [19,36,37]. In a study placing a set of enquiries at DI centers, correct answers were given in 5–90% depending on the topic. Of 20 factors regarding the staff’s professional background and the characteristics of the DI center, none influenced the correctness of the answer [19]. In another study, a procedure manual describing how DI should be performed ensured more accurate results [36].

The quality of DI also depends on the way it is presented. This has been evaluated by previous studies, and criteria for structural quality have been defined [17,18]. In our study, an increase in the number of fulfilled structural requirements can be seen over the years, which was confirmed in the multivariate logistic regression. Most likely, this was a learning effect from repeated annual tests. However, since all data regarding participants were anonymized, we cannot determine how often they took part and if repeated participation was responsible for a learning effect. The organization of DI within the hospital pharmacy also had an impact on the presentation of references and conclusion/recommendation.

We expected that several quality measures might improve the results. Surprisingly, the DI and ward experience of the pharmacist answering the test enquiry had no impact. Documentation databases offer several advantages from a guided workflow and ensuring that the recommended process of answering DI enquiries is followed to the possibility of searching for information stored in previous answers [6,38]. However, no impact was seen.

Second look is a recommended quality measure for DI [2,3,4]. This was the only quality measure to consistently show an impact on content-related and structural quality.

Our study has several limitations. The UKMI enquiry level, the number of participating hospital pharmacies, and the proportion of hospital pharmacies with a DI center differed in the test years, which might have had an impact on our statistical analysis. In addition, comparisons across the years were complicated by a different number of predefined essential information. For this reason, we analyzed this issue in two ways, leading to comparable results: for the presentation of all expected essential information and, in addition, for the percentage of essential information achieved. However, as a result of our study, the UKMI enquiry level and topic were found to impact the quality of the answers. All information on the organization of DI in a hospital pharmacy and implemented quality measures was self-reported, thus, we cannot rule out positive or negative bias due to incorrect reporting. As mentioned above, due to the irreversible anonymized data, we cannot say how often a hospital pharmacy participated or the same person in a participating hospital pharmacy answered the question in several years. Also, we did not analyze the number and nature of references used in the presented answers. This might have had an additional impact on the quality and will be the focus of following studies.

## 5. Conclusions

The quality of DI provided by hospital pharmacies depends on the complexity of the enquiry and the organization of the professional task of DI within the hospital pharmacy. Specialized DI center achieved better results in the content-related and structural quality of answers and their presence should be encouraged. In addition, second look as a quality measure improved results, and should be implemented as a standard measure. Furthermore, several areas for improvement could be identified by annual DI tests with fictitious enquiries, which will be addressed in academic and professional training.

## Figures and Tables

**Table 1:** Predefined structural requirements (n = 10) *.

Answer corresponding to question
Logical organization of answer
Conclusion/recommendation presented
Conclusion/recommendation logically deduced of presented information
References given
References presented in a way they can be tracked/checked
Correct grammar and spelling
Absence of unclear or misleading information
Good readability and understandability
Length of answer appropriate

* based on [17,18].

**Table 2:** Characteristics of annual quality assessments of drug information 2017–2021.

Test Year	2017	2018	2019	2020	2021
Topic	Contraindication/drug choice	Preoperative drug management	Drugs in lactation	Overdose/toxicology	Adverse drug reaction
Enquiry level [25]	2	3	2	2	3
No. of predefined essential information	3	5	2	3	5
No. of predefined additional useful information	6	8	8	10	6

**Table 3:** Characteristics of participants and results on content-related and structural requirements on test enquiries. Percentages presented refer always to the number of participants of the respective year or years 2017–2021, respectively.

Year	2017	2018	2019	2020	2021	2017–2021
No. of participants [n (%)]	45 (100)	71 (100)	79 (100)	118 (100)	122 (100)	435 (100)
Characteristics of participants						
*No. of pharmacists [median (range)]*	6 (2–26)	5.5 (1–29)	6.5 (2–33)	6 (1–34)	6 (2–25)	6 (1–34)
*DI Organization* ^1^						
*DI center [n (%)]*	11 (25)	17 (24)	18 (23)	19 (16)	16 (13)	81 (19)
*Pharmacist per day [n (%)]*	5 (11)	5 (7)	10 (13)	4 (3)	4 (3)	28 (6)
*On routine [n (%)]*	29 (64)	49 (69)	47 (60)	93 (79)	87 (71)	305 (70)
*Unknown [n (%)]*	0	0	4 (5)	2 (2)	15 (12)	21 (5)
*No. of quality measures* ^2^ *[median (range)]*	3 (1–4)	2 (0–4)	3 (1–4)	2.5 (0–4)	3 (0–4)	3 (0–4)
*Level DI experience of the answering pharmacist* ^3^ *[median (range)]*	4 (1–4)	3 (1–4)	3 (0–4)	3 (1–4)	3 (1–4)	3 (0–4)
Content-related results on test inquiry						
*No. of participants with all essential information [n (%)]*	28 (62)	22 (31)	74 (94)	88 (75)	8 (7)	220 (49)
*No. of optional contents [median (range)]*	4 (0–7)	4 (0–8)	5 (2–8)	3 (0–8)	2 (0–6)	3 (0–8)
*No. of participants with irrelevant information [n (%)]*	11 (24)	20 (28)	19 (24)	53 (45)	35 (29)	138 (32)
*No. of participants with misleading or wrong information [n (%)]*	8 (18)	11 (15)	35 (44)	15 (13)	14 (12)	83 (19)
Results on structural requirements for test enquiry (max. 10)						
*No. of fulfilled structural requirements [median (range)]*	7 (1–10)	8 (2–10)	8 (2–9)	8 (2–10)	9 (2–10)	8 (1–10)
*Length of answer appropriate [n (%)]*	27 (60)	41 (58)	54 (68)	78 (66)	84 (69)	284 (65)
*No. of answers with named references [n (%)]*	36 (80)	59 (83)	77 (97)	110 (93)	115 (94)	397 (91)
*No. of answers with trackable references [n (%)]*	21 (47)	50 (70)	53 (67)	98 (81)	107 (88)	327 (75)
*No. of answers presenting conclusion/recommendation [n (%)]*	27 (60)	40 (56)	63 (80)	70 (59)	107 (88)	307 (71)

^1^ DI organization: DI center = drug information center in hospital pharmacy; pharmacist per day = per day a pharmacist is responsible for DI tasks; on routine = DI has to be performed in addition to other routine tasks in the pharmacy like compounding or dispensing; unknown = information on DI organization not given; ^2^ quality measures (max. 4): second look; answering pharmacist has experience on ward; use of a DI documentation system; additional measures; ^3^ DI experience of the answering pharmacist: (level 1: <1 year; level 2: 1–3 years; level 3: 3–5 years; level 4: >5 years).

**Table 4:** Multivariate logistics regression investigating the influence of parameters on content-related quality of answers: (A) all essential information presented and (B) percentage of essential information fulfilled. Statistically significant results at the 5% level are given in bold.

Parameter	Coefficient	OR	95% CI	*p*
**A: All essential information presented**				
Year 2017	1.45	4.28	0.98–18.6	0.05
Year 2018	−1.40	**0.25**	**0.11–0.56**	**<0.01**
Year 2019	2.10	**8.19**	**2.69–24.9**	**<0.01**
Year 2020	0.69	1.99	0.92–4.31	0.08
Year 2021	−3.13	**0.04**	**0.01–0.11**	**<0.01**
DI center	reference	-	-	-
Pharmacist responsible per day	−0.29	0.74	0.21–2.67	0.65
DI in addition to routine tasks	−1.04	**0.35**	**0.15–0.83**	**0.02**
No. of Pharmacists	0.01	1.01	0.94–1.06	0.88
Experience in DI	−0.09	0.91	0.72–1.15	0.43
Second look	0.38	1.47	0.84–2.56	0.17
Experience on ward	−0.15	0.86	0.45–1.62	0.64
Documentation system	−0.01	0.99	0.54–1.80	0.98
**B: Percentage of essential information fulfilled**				
Year 2017	0.86	**2.38**	**2.13–2.65**	**<0.01**
Year 2018	−0.03	0.96	0.89–1.04	0.34
Year 2019	0.13	**1.15**	**1.07–1.23**	**<0.01**
Year 2020	0.08	**1.08**	**1.01–1.15**	**0.02**
Year 2021	−0.15	**0.86**	**0.80–0.91**	**<0.01**
DI center	Reference	-	-	-
Pharmacist responsible per day	−0.02	0.97	0.89–1.06	0.57
DI in addition to routine tasks	−0.06	**0.93**	**0.89–0.99**	**0.04**
No of Pharmacists	0.01	1.00	0.99–1.01	0.58
Experience DI	−0.01	0.99	0.98–1.01	0.68
Second look	0.01	1.01	0.97–1.05	0.58
Experience on ward	−0.01	0.99	0.95–1.56	0.80
Documentation system	0.01	1.00	0.96–1.04	0.98

**Table 5:** Multivariate logistics regression investigating the influence of parameters on the quality of answers regarding structural requirements: (A) references given; (B) references given in a trackable way; and (C) conclusion presented. Statistically significant results at the 5% level are given in bold.

Parameter	Coefficient	OR	95% CI	*p*
**A: References given**				
Year 2017	1.57	4.82	0.34–67.2	0.24
Year 2018	0.31	1.36	0.48–3.81	0.56
Year 2019	2.40	**10.99**	**2.13–56.7**	**<0.01**
Year 2020	1.45	**4.27**	**1.42–7.33**	**<0.01**
Year 2021	1.68	**5.39**	**1.63–17.8**	**<0.01**
DI center	reference	-	-	-
Pharmacist responsible per day	−3.15	**0.04**	**0.01–0.45**	**<0.01**
DI in addition to routine tasks	−2.04	0.13	0.01–1.15	0.07
No of Pharmacists	0.03	1.03	0.92–1.15	0.61
Experience DI	0.20	1.22	0.89–1.66	0.21
Second look	0.68	1.97	0.91–4.29	0.09
Experience on ward	0.74	2.09	0.94–2.29	0.07
Documentation system	0.08	1.08	0.49–2.37	0.85
**B: References given in trackable way**				
Year 2017	−0.93	0.39	0.10–1.56	0.18
Year 2018	1.06	**2.89**	**1.30–6.54**	**0.01**
Year 2019	0.86	**2.36**	**1.05–5.28**	**0.04**
Year 2020	1.65	**5.19**	**2.34–11.5**	**<0.01**
Year 2021	2.13	**8.45**	**3.52–20.2**	**<0.01**
DI center	reference	-	-	-
Pharmacist responsible per day	−0.69	0.50	0.17–1.51	0.22
DI in addition to routine tasks	−0.26	0.77	0.33–1.79	0.54
No of Pharmacists	0.05	1.05	0.98–1.12	0.11
Experience DI	−0.04	0.96	0.78–1.18	0.11
Second look	0.65	**1.92**	**1.16–3.17**	**0.01**
Experience on ward	0.38	1.46	0.93–2.57	0.19
Documentation system	0.26	1.30	0.76–2.22	0.32
**C: Conclusion presented**				
Year 2017	−0.01	0.99	0.26–3.71	0.98
Year 2018	−0.19	0.82	0.37–4.07	0.64
Year 2019	1.00	**2.72**	**1.55–6.46**	**0.02**
Year 2020	−0.01	0.99	0.43–2.09	0.98
Year 2021	1.52	**4.59**	**1.94–10.86**	**<0.01**
DI center	Reference	-	-	-
Pharmacist responsible per day	−0.58	0.56	0.18–1.71	0.31
DI in addition to routine tasks	−0.58	0.56	0.25–1.24	0.15
No of Pharmacists	0.06	**1.06**	**1.01–1.13**	**0.04**
Experience DI	0.16	1.17	0.96–1.42	0.11
Second look	0.39	1.47	0.92–2.36	0.11
Experience on ward	−0.45	0.64	0.36–1.12	0.11
Documentation system	0.22	1.24	0.75–2.05	0.39

## Data Availability

Data are available from the authors on reasonable request.

## Supplementary Materials

The following supporting information can be downloaded at: https://www.mdpi.com/article/10.3390/pharmacy12040109/s1, Table S1: Test enquiries of the annual DI quality assessment and predefined, requested essential information [12,26,27,28,29].
