# Supplementary material for: Factors Determining Quality of Drug Information by Hospital Pharmacies—Results from Five-Year Annual Quality Assessment
Source: Pharmacy (Basel). 2024 Jul 13;12(4):109. doi: 10.3390/pharmacy12040109 (PMC11270217; doi:10.3390/pharmacy12040109)
Supplement: Supplementary file 1 [file pharmacy-12-00109-s001.zip › pharmacy-3063672-supplementary.pdf]

# Factors Determining Quality of Drug Information by Hospital Pharmacies—Results from Five-Year Annual Quality Assessment

**Table S1.** Test enquiries of the annual DI quality assessment and predefined, requested essential information [12, 26-29].

| Year | Test enquiry | requested essential information |
|---|---|---|
| 2017 | A physician at your hospital is calling. He wants to prescribe dexketoprofen for trigeminal neuralgia. The patient has renal impairment. Which dosage should be used?<br>Female patient, aged 77 years, admitted to surgical unit for ablation of benign intestinal polyps. Renal function (CKD- EPI): eGFR=35 mL/min/1.73 m². Episode of trigeminal neuralgia 25 years ago; patient does not know how it was treated then. Symptoms re-appeared lately after being absent for many years. She had tried ibuprofen which did not help. Additional medication: ramipril/hydrochlorothiazide 5 mg/25 mg, levothyroxine 75 µg, atorvastatin 20 mg | (1) Dexketoprofen contraindicated in renal impairment<br>(2) Dexketoprofen is not a treatment of choice for trigeminal neuralgia<br>(3) Suggestion for proceeding further (eg, reference to guideline trigeminal neuralgia, carbamazepine as drug of first choice or another therapeutic option mentioned) |
| 2018 | An enquiry is received via e-mail from a doctor in neurosurgery. A female patient with bipolar disease is successfully treated for a long time with lithium, duloxetine and risperidone. In addition, she is taking green-tea-capsules as self-medication. A neurosurgical intervention is planned in two weeks. Please advice on the preoperative handling of the current medication! | (1) Lithium: stop 48h prior to surgery OR specific explanation how to handle an ongoing lithium intake during surgery<br>(2) do not stop duloxetine abruptly<br>(3) consider increased bleeding risk while taking duloxetine in the context of a neurosurgical intervention<br>(4) do not stop risperidone<br>(5) advice on the preoperative handling of green-tea-capsules |
| 2019 | A 38-year old nursing mother (child 3 month old, fully breastfed) with proven heparin-induced thrombocytopenia needs thromboprophylaxis for a planned surgical intervention on her foot. Fondaparinux was used during former surgery, however, the patient requests an oral drug and still wants to breastfeed. Can one of the new direct acting oral anticoagulants be used? | (1) Fondaparinux can be used safely while breastfeeding<br>(2) new direct oral anticoagulants are unsafe during breastfeeding |
| 2020 | A doctor from the geriatric ambulatory unit is calling. An older female patient, sometimes confused, underweighted, took an unknown | (1) no harm is to be expected by the omeprazole overdose |

| | | |
|---|---|---|
| | number of omeprazole 40 mg yesterday, maximum 10 tablets according to her accompanying daughter. The patient is 82 years old, has renal impairment (last measure 37 ml/min/1,73 m²). Her further medication is acetylic salicylic acid 100 mg once daily, cholecalciferol 20.000 IE and alendronate 70 mg once weekly, and an unknown multivitamin preparation. The patient herself does not communicate much, so far she seems asymptomatic. What consequences are to be expected? Are specific measures recommended, e.d. haemodialysis? | (2) possible symptoms of overdose are mentioned<br>(3) haemodialysis is not indicated |
| 2021 | A doctor of the ambulatory psychiatric unit is calling. A male patient, 38 years, schizophrenia successfully and controlled by TDM treated with clozapine and aripiprazole wishes to father a child. Please advice on possible fertility impairment and hormonal effects of the medication! | (1) no evidence for fertility impairment under clozapine<br>(2) no evidence for fertility impairment under aripiprazole<br>(3) possible impact on testosterone level discussed<br>(4) possible impact on prolactin level discussed<br>(5) both drugs are not genotoxic, mutagenic or cancerogenic |
